# The Effect of Different Levels of Cu, Zn and Mn Nanoparticles in Hen Turkey Diet on the Activity of Aminopeptidases

**DOI:** 10.3390/molecules23051150

**Published:** 2018-05-11

**Authors:** Artur Jóźwik, Joanna Marchewka, Nina Strzałkowska, Jarosław Olav Horbańczuk, Małgorzata Szumacher-Strabel, Adam Cieślak, Paulina Lipińska-Palka, Damian Józefiak, Agnieszka Kamińska, Atanas G. Atanasov

**Affiliations:** 1 Institute of Genetics and Animal Breeding, Polish Academy of Sciences, 05-552 Jastrzębiec, Poland; n.strzalkowska@ighz.pl (N.S.); j.horbanczuk@ighz.pl (J.O.H.); lipinskap.igab.pas@gmail.com (P.L.-P.); atanas.atanasov@univie.ac.at (A.G.A.); 2 Department of Animal Nutrition and Feed Management, Poznań University of Life Sciences, 60-637 Poznań, Poland; mstrabel@up.poznan.pl (M.S.-S.); adam.cieslak@up.poznan.pl (A.C.); damjo@up.poznan.pl (D.J.); 3 Department of Family Studies, Cardinal Stefan Wyszynski University in Warsaw, 01-815 Warszawa, Poland; agnieszka.kaminska73@wp.pl; 4 Department of Pharmacognosy, University of Vienna, 1090 Vienna, Austria

**Keywords:** nanoparticles, turkey, aminopeptidases, poultry meat, nutrition, supplementation, lysosomal enzymes

## Abstract

The aim of the study was to estimate the influence of the different levels of Cu, Zn, and Mn nanoparticles on the activity of aminopeptidases in turkey. An experiment was carried out on 144 turkey hen Hybrid Converter. The birds were divided into groups given standard- and nanoparticle-supplementation of different level of copper (Cu 20, 10, 2 mg/kg), zinc (Zn 100, 50, 10 ppm), and manganese (Mn 100, 50, 10 ppm), covering respectively 100%, 50%, and 10% of the physiological demands for those minerals in the diet. The activity of aminopeptidases (alanyl: AlaAP, leucyl: LeuAP and arginyl: ArgAP) after supplementation of minerals was determined in the breast and thigh turkey muscle. The strongest effect of interaction among minerals supplementation form and dose on the activity levels of the aminopeptidases in thigh muscle was observed for nano-Cu already at the lowest dose of 2 mg/kg. In this dose (covering 10% of the birds’ demand) nano form of supplementation significantly increased the activity of Ala-, Leu-, and ArgAP (877, 201, and 719, respectively), compared to standard form of supplementation (461, 90.5, and 576, respectively). In turn, in breast muscle, after supplementation covering 10% of the demand with the nano-Cu, nano-Zn, and nano-Mn compared to the standard form, we did not observe any significant difference in the activity levels of any of the investigated aminopeptidases, except for AlaAP under Zn supplementation. Supplementation with the 20 mg/kg of Nano-Cu (100% of demand) and with 10 mg/kg of Nano-Cu (50% of demand) inhibited the activity of all of the three aminopeptidases in thigh muscle. Supplementation of the minerals in nano form into the diet, especially of Cu and Zn in the dose covering 10% of the demand is relevant to maintain homeostasis in turkey muscles, as indicated by the activity of the aminopeptidases.

## 1. Introduction

Poultry meat belongs to a group of high-quality food products because of its valuable dietetic and nutritional properties, providing the necessary proteins and trace minerals into the human diet [1,2,3]. Feeding management of poultry species has been identified as one of the major factors assuring birds health and high levels of welfare [4,5], therefore affecting their final performance [6].

The maintenance of cellular homeostasis involves lysosomal enzymes, including aminopeptidases, responsible not only for the accumulation and circulation of proteins and microelements in the animal body. Changes in the aminopeptidase activity affect the bioavailability of the nutrients from the feed, composition of the muscle, and—in case of production animals—the final product quality [7]. Trace elements like Cu, Zn, or Mn are essential bioactive compounds, influencing the functions of the organism and regulate the activity of the enzymes and therefore are important for maintaining cell homeostasis in muscle [8,9,10].

Zinc, copper, and manganese are significant supplements in commercial poultry diets, and are provided traditionally in feed using inorganic salts such as oxides and sulfates. The availability of minerals from feed materials of plant origin, as well as from traditional inorganic sources—i.e., oxides, sulphates, or carbonates—is relatively low, while the requirements of modern poultry production for microelements are very high [11]. One of the newest delivery methods of the micronutrients in poultry feeding is nanotechnology recognized as an innovative field of science investigating molecules and molecular structures with nanometric size [12]. The delivery method of abovementioned microelements in the birds’ diet might have an effect on their bioavailability [13], as well as their form and dosage may affect changes in the activity of the aminopeptidases.

It is also crucial to avoid the oversupplementation of the Cu, Mn, and Zn in the poultry diets. This is important aspect since the oversupplementation of those minerals negatively affects the animals’ performance [12,14]. Thus, the essential minerals must be provided in the diet at concentrations sufficient optimum response and the dietary intake of other minerals must be low enough to provide complete safety for both the animals and also the human population which will consume animal products including meat [15]. In the current literature, there is a shortage of information about effect of addition of mineral nanoparticles in bird’s diet on the activity of lysosomal enzymes including aminopeptidases, which is an important indicator of the trace minerals retention and bioavailability and protein turnover in the bird’s body.

The aim of this study was to evaluate the influence of supplementation of the diet with different levels of nanoparticles and traditional form—i.e., Cu, Zn, and Mn—on the activity of the selected, most indicative aminopeptidases: alanyl: AlaAP, leucyl: LeuAP, and arginyl: ArgAP in turkey meat.

## 2. Materials and Methods

### 2.1. Birds and Diet

The experiment was conducted on a turkey farm located in the north-eastern part of Poland, using 144 turkey hen hybrid converter (*n* = 8 birds).

The birds were kept in pens, each with an area of 3.7 m^2^ with a wood chip bedding. The stocking density was at the level of 4.8 birds/m^2^ for the first 6 weeks and 3.2 birds/m^2^ from seven weeks onward until the end of production cycle (98 days). The experimental building was equipped with automatically controlled lighting, heating, and ventilation system. Light programs and temperature were in accordance with the recommendations of hybrid turkeys (Hendrix Genetics, Boxmeer, The Netherlands). Birds had ad libitum access to drinking water and feed mixtures. All birds were fed according to the diet shown in the Table 1.

The vitamin premix was added to the feed as a second step of the diet preparation. It covered the total (100%) of the demand for the two minerals not being a treatment, all provided in a standard form, while the treatment mineral was present in the premix in the form and dose respective to the specific treatment described below.

In order to test the effect of mineral supplementation form in turkey feeding, the birds were divided into two groups. Control groups received a standard form of the studied minerals, while the test groups received the mineral in the nanoparticles form. The birds in both groups were supplemented with different levels of copper (Cu 20, 10, 2 mg/kg), manganese (Mn 100, 50, 10 ppm), and zinc (Zn 100, 50, 10 ppm) respectively covering 100%, 50%, and 10% of the physiological demands for those minerals in the diet. The nanoparticles of Cu, ZnO, and Mn_2_O_3_ were obtained as a powder from Sky Spring Nanomaterials Inc. (Houston, TX, USA). They were applied either in the form of a powder or of a colloid prepared based on the miliQ water. The treatments receiving standard form of the minerals included copper in the form of CuSO_4_, zinc in the form of ZnO and manganese, as MnO.

### 2.2. Sampling

The turkey breast (*M. pectoralis*) and thigh (*M. iliotibialis*) muscle meat samples were collected immediately after slaughter (max 40 min) and frozen in liquid nitrogen (−80 °C). Then, the samples were homogenized in 0.1 M phosphate buffer (pH 7.0) with 0.1% Triton X100 according to the modified method of [16].

### 2.3. Aminopeptidases Assay Procedure

The activities of alanyl-aminopeptidase (AlaAP-EC 3.4.11.2), leucylaminopeptidase, and arginyl-amino-peptidase (ArgAP-EC 3.4.11.6) were determined by the method of [17] in obtained supernatants. The activity of AlaAP, LeuAP, and ArgAP was expressed in nmol/mg protein/h and were measured as Fast Blue BB salt (4-benzoyloamino-2, 5-diethoxybenzene-diazinium chloride) derivatives at 540 nm with use of spectrometer Cary Varian 50Bio (Santa Clara, CA, USA).

### 2.4. Statistical Analysis

A generalized linear mixed model analysis was performed on all measured parameters: AlaAP, LeuAp, and ArgApP including supplementation form, dose, and their interaction as fixed factors. A separate model was run for each of the minerals: Cu, Mn, and Zn in each of two types of muscle—breast and thigh—the validity of the models was tested by using Akaike’s information criterion. PROC GLIMMIX of SAS v 9.3 [18] including the Tukey adjustment option was used to conduct the analysis. The least square means for all significant effects in the models (*p* ≤ 0.05) were computed using the LSMEANS option.

## 3. Results

The activities of alanyl-aminopeptidase (AlaAP-EC 3.4.11.2), leucylaminopeptidase (LeuAP 3.4.11.1) and arginyl-amino-peptidase (ArgAP-EC 3.4.11.6), as subjected to the different levels of supplementation in nano- and standard form were analyzed in turkey breast and thigh muscle. Obtained results have been presented respectively in Table 2, Table 3 and Table 4.

The strongest effect of interaction between Cu supplementation form and dose on the activity levels of all aminopeptidases was observed in thigh muscle already at the lowest dose 2 mg/kg. The lowest dose of Cu (10% of the demand) in nanoform increased the activity of Ala-, Leu-, and ArgAP significantly higher than standard form, as well as of all other applied treatments (Table 2). Supplementation with the 20 mg/kg of Nano-Cu (100% of demand) and with 10 mg/kg of Nano-Cu (50% of demand) compared to the 2 mg/kg of Nano-Cu, clearly inhibited the activity of all of the three aminopeptidases (Table 2).

In turn, in breast muscle, after supplementation covering 10% of the demand with the nano-Cu compared to the standard form, we did not observe any significant difference in the activity levels of any of the investigated aminopeptidases. In case of higher doses of Cu supplementation, at the level of 50% provision of the nano-Cu in this muscle increased the LeuAP activity compared to standard form, while at the level of 100% nano-Cu decreased the activity of AlaAP compared to standard form (Table 2).

As presented in the Table 3, 2 mg/kg (10% of the demand) of Nano-Zn increased the LeuAP activity in thigh muscle, comparably to the 10 mg/kg of Nano-Zn (50% of demand) but not of the 20 mg/kg of Nano-Zn (100% of demand). Nano-Zn at the lowest level (10% of the demand) significantly increased the activity of LeuAP compared to the activity when the same dose of this mineral in the standard form was provided.

In the breast muscle, provision of the Nano-Zn at the level of 2 mg/kg caused the AlaAP activity decrease when compared to provision of this mineral in the same dose in the standard form (Table 3), as well as when compared to provision of the Nano-Zn at the level of 20 mg/kg. In turn, provision of Zn in the standard form on the level of 20 and 10 mg/kg increased the activity of ArgAP when compared to the same doses of nano-Zn.

No effect of interaction between Mn supplementation form and dose on the activity levels of any aminopeptidase neither in thigh nor in breast muscles was observed (Table 4). A medium dose (50%) of Mn supplementation significantly decreased activity of ArgAP in thigh muscle (Table 4), compared to the lowest dose (10% of the demand). Dose of Mn covering 50% of the demand decreased the activity of LeuAP compared to dose covering 10% and 100% of the demand.

## 4. Discussion

Obtained results in our study demonstrated that supplementation of minerals, especially Cu in the nano form into turkey diet significantly influenced the aminopeptidases activity. It should be noted that changes in the activity of the aminopeptidases are conditioned by different factors covering the dosage and form of supplementation of minerals like Cu, Zn, or Mn which are essential bioactive compounds [14,15]. For example copper and zinc are necessary for proper metabolism and for activity of vitally important enzymes like aminopeptidases, although it is toxic when in excess. Thus, their supply and uptake have to be regulated [19]. In our study as already the lowest level of Cu (2 mg/kg—10% of the demand) leads to significantly high activity of aminopeptididases, especially in nano form in thigh muscles as compared to traditional form which means that the former is more bioavailable in relation to standard form. It is interesting that, in the case of Cu at the lowest level of dosage (10% in demand), significant differences between tight and breast muscle were observed. It should be noted that these muscles vary in morphology, biochemically and functionally. Muscles of turkey are made of two types of fibers: fast-twitch fibers contract quickly for infrequent bursts of activity, while slow twitch muscle fibers fire more slowly than fast twitch fibers, as well as they fatigue slower [9,20]. Turkeys use their legs continuously, which is why thighs are darker type of meat, whereas their more inactive breast muscles become white meat. Those muscles consist primarily of large number of mitochondria, with changing activity of oxidative and degradative enzymes, affecting protein synthesis [20,21]. Initially, the degradation of major myofibrillar proteins results in the formation of intermediate polypeptides that will be degraded to small peptides by the action of peptidases and finally, to free amino acids by the activity of aminopeptidases [22]. Both peptides and amino acids are responsible for the characteristic flavor of meat and meat products [23]. The administration of especially copper and also zinc in the form of nanoparticles at doses of 50 and 100% inhibits the activity of aminopeptidases and can be accumulated in the body and might decrease the bird’s performance. Similar effects was observed by Marchi et al., 2004. Oversupplementation of Cu or Zn in the diet lead to the deposition of these metals and affect growth of cells and tissues [24,25,26]. The observed inhibition of aminopeptidases in present study at a dose of 100% and 50% may be associated with too high physiological load of muscles tissue with this element. Too much zinc loading leads to disturbance of cellular homeostasis. Kukica studies [24] showed that the lysosomal system, including aminopeptidases, has a cytoprotective role in exposure to zinc and, thus, increases autophagy and lysosomal enzyme activity. The observed inhibition of aminopeptidase activity in some muscles may adversely affect cellular homeostasis. Other studies have shown that inhibiting the activity of aminopeptidases in lysosomes can increase the content of zinc in the mitochondria, leading to apoptosis [27].

## 5. Conclusions

Among three used minerals (Cu, Zn, Mn) the strongest effect of interaction of supplementation form and dose on the activity levels of the aminopeptidases was observed in case of Cu—already at the lowest dose 2 mg/kg. The activity of Ala-, Leu-, and Arg-AP in nanoform increased significantly more than in standard form in case of thigh muscle. In turn, in breast muscle, after Cu, Zn, and Mn supplementation we did not observe any significant difference in the activity levels of any of the investigated aminopeptidases, except for AlaAP under Zn suppelentation. Supplementation with the 20 mg/kg of Nano-Cu (100% of demand) and with 10 mg/kg of Nano-Cu (50% of demand) clearly inhibited the activity of all of the three aminopeptidases, which can even lead to accumulation of the investigated minerals in the body and can affect negatively the performance in turkey production.

We concluded that supplementation of the minerals in nano form into the turkey diet, especially of Cu and Zn in the dose covering 10% of the demand is relevant to maintain homeostasis in turkey muscles, as indicated by the activity of the aminopeptidases.

## Figures and Tables

**Table 1:** Feed and nutrient composition of turkey hens at different ages.

		Diet and Age (Days)		
		Starter	Grower	Finisher
Nutritional value		1–42 days	43–70 days	71–98 days
Protein	%	26.50	23.00	18.50
Fiber	%	3.40	3.98	3.57
Fat	%	4.23	7.16	7.37
Amino acids				
Arginine	%	1.76	1.52	1.18
Lysine	%	1.74	1.50	1.17
Methionine	%	0.71	0.57	0.45
Methionine + cysteine	%	1.13	0.95	0.78
Threonine	%	1.05	0.93	0.68
Tryptophan	%	0.32	0.29	0.22
Minerals				
Ca	%	1.15	1.05	0.65
P	%	0.55	0.45	0.30
Na	%	0.15	0.13	0.13
Energy	kcal/kg	2750	2950	3100

**Table 2:** The effect of Cu supplementation in three doses (100%, 50%, and 10% of the requirement) and two forms (nanonaparticles and standard) into hen turkey diet on the AlaP, LeuAP, and ArgAP activity levels (mean ± SE) in breast and thigh muscle.

	Breast Muscle			Thigh Muscle		
Group *	AlaAP	LeuAP	ArgAP	AlaAP	LeuAP	ArgAP
100S	658.4 ^a^ ± 17.8	133.4 ^b,c^ ± 4.8	402.8 ^b,c^ ± 14.2	556.4 ^c,d^ ± 22.5	119.4 ^b^ ± 5.9	387.5 ^c,d^ ± 15.4
100N	411.2 ^b^ ± 27.7	93.2 ^d^ ± 6.6	354.3 ^c^ ± 19.6	510.4 ^c,d^ ± 11.9	147.7 ^b^ ± 4.9	378.8 ^c,d^ ± 12.9
50S	680.9 ^a^ ± 36.7	114.2 ^c,d^ ± 4.9	500.5 ^a^ ± 14.9	712.6 ^b^ ± 8.7	132.4 ^b^ ± 4.5	417.2 ^c^ ± 13.7
50N	628.9 ^a^ ± 32.2	164.4 ^a,b^ ± 12.4	413.5b ^c^ ± 25.3	602.2 ^c^ ± 44.8	74.6 ^c^ ± 3.9	327.8 ^d^ ± 13.0
10S	599.9 ^a^ ± 16.1	197.4 ^a^ ± 6.5	450.7 ^a,b^ ± 13.9	461.1 ^d^ ± 22.6	90.5 ^c^ ± 8.9	576.8 ^b^ ± 24.9
10N	698 ^a^ ± 9.7	179.9 ^a^ ± 13.8	428.9 ^a,b^ ± 9.7	877.7 ^a^ ± 20.2	201.0 ^a^ ± 10.1	719.1 ^a^ ± 33.0
**Suppl. form effect**						
Nanoparticles	579.4 ^b^ ± 29	145.9 ± 10	398.9 ^b^ ± 12.6	666.1 ^a^ ± 37.1	143.9 ^a^ ± 11.5	481.7 ± 39.2
Standard	646.4 ^a^ ± 15.7	148.3 ± 8	451.3 ^a^ ± 11.5	576.7 ^b^ ± 24.0	114.1 ^b^ ± 5.2	460.5 ± 20.2
**Dose effect**						
100%	534.8 ^b^ ± 35.7	113.3 ^c^ ± 6.5	378.6 ^b^ ± 13.3	533.4 ^b^ ± 13.6	133.6 ^a^ ± 5.2	383.2 ^b^ ± 9.8
50%	654.9 ^a^ ± 24.5	139.3 ^b^ ± 9.1	456.9 ^a^ ± 18.1	661.1 ^a^ ± 25.2	105.4 ^b^ ± 8.2	375.5 ^b^ ± 15.0
10%	648.9 ^a^ ± 15.6	188.7 ^a^ ± 7.7	439.8 ^a^ ± 8.7	669.4 ^a^ ± 55.7	145.7 ^a^ ± 15.7	647.9 ^a^ ± 27.1
	*p*-value					
**Source of variation**						
Suppl. Form	0.0023	0.74	0.0005	<0.001	<0.001	0.38
Dose	<0.001	<0.001	<0.001	<0.001	<0.001	<0.001
Suppl. form × Dose	<0.001	<0.001	0.17	<0.001	<0.001	<0.001

* 100S: 100% of the dose in a standard form; 100N: 100% of the dose in a nanoparticle form; 50S: 50% of the dose in a standard form; 50N: 50% of the dose in a nanoparticle form; 10S: 10% of the dose in a standard form; 10N: 10% of the dose in a nanoparticle form; ^a–d^ Means in the same column with different letters are significantly different (*p* < 0.05) separately between the group, between the species and between the additives.

**Table 3:** The effect of Zn supplementation in three doses (100%, 50%, and 10% of the requirement) and two forms (nanonaparticles and standard) into hen turkey diet on the AlaP, LeuAP, and ArgAP activity levels (mean ± SE) in breast and thigh muscle.

	Breast Muscle			Thigh Muscle		
Group *	AlaAP	LeuAP	ArgAP	AlaAP	LeuAP	ArgAP
100S	243.3 ^a^ ± 21.7	40.7 ± 3.7	136.6 ^a^ ± 12.0	253.1 ± 9.8	80.2 ^a^ ± 6.4	263.3 ± 25.8
100N	164.6 ^b^ ± 8.5	35.4 ± 3.6	104.4 ^b^ ± 6.7	235.9 ± 6.0	71.5 ^a^ ± 6.8	289.9 ± 38.8
50S	205.5 ^a^ ± 35.5	43.0 ± 5.3	138.7 ^a^ ± 21.9	187.5 ± 5.9	55.0 ^b^ ± 1.4	190.5 ± 4.8
50N	157.8 ^b,c^ ± 16.8	34.0 ± 7.0	103.7 ^b^ ± 10.7	166.7 ± 6.2	53.6 ^b^ ± 2.4	168.5 ± 5.2
10S	162.8 ^b^ ± 7.2	27.2± 1.7	103.5 ^b^ ± 4.8	159.4 ± 23.4	56.3 ^b^ ± 7.7	182.3 ± 23.0
10N	138.5 ^c^ ± 14.8	30.5 ± 6.0	106.9 ^b^ ± 15.3	189.9 ± 9.5	76.2 ^a^ ± 1.8	212.7 ± 19.8
**Suppl. form effect**						
Nanoparticles	153.6 ^b^ ± 7.9	33.3 ± 3.0	105.0 ± 6.0	197.5 ± 9.5	67.1 ± 3.7	223.7 ± 20.1
Standard	203.9 ^a^ ± 16.1	36.9 ± 2.9	126.3 ± 9.1	200.0 ± 14.2	63.8 ± 4.6	212.0 ± 15.2
**Dose effect**						
100%	203.9 ^a^ ± 18.4	38.1 ± 2.6	120.5 ± 8.8	244.5 ^a^ ± 6.2	75.9 ^a^ ± 4.6	276.6 ^a^ ± 22.2
50%	181.7 ^a,b^ ± 20.3	38.5 ± 4.4	121.2 ± 13.0	177.1 ^b^ ± 5.6	54.3 ^b^ ± 1.3	179.5 ^b^ ± 5.3
10%	150.6 ^b^ ± 8.9	28.8 ± 2.9	105.2 ± 7.4	174.5 ^b^ ± 13.0	66.2 ^a,b^ ± 5.2	197.5 ^b^ ± 15.1
	*p*-value					
**Source of variation**						
Suppl. Form	0.0061	0.37	0.06	0.79	0.44	0.54
Dose	0.047	0.11	0.41	<0.001	0.0021	0.001
Suppl. form × Dose	0.04	0.44	0.04	0.08	0.03	0.46

* 100S: 100% of the dose in a standard form; 100N: 100% of the dose in a nanoparticle form; 50S: 50% of the dose in a standard form; 50N: 50% of the dose in a nanoparticle form; 10S: 10% of the dose in a standard form; 10N: 10% of the dose in a nanoparticle form; ^a–c^ Means in the same column with different letters are significantly different (*p* < 0.05) separately between the group, between the species and between the additives.

**Table 4:** The effect of Mn supplementation in three doses (100%, 50%, and 10% of the requirement) and two forms (nanonaparticles and standard) into hen turkey diet on the AlaP, LeuAP, and ArgAP activity levels (mean ± SE) in breast and thigh muscle.

	Breast Muscle			Thigh Muscle		
Group *	AlaAP	LeuAP	ArgAP	AlaAP	LeuAP	ArgAP
100S	888.7 ± 11.1	130.0 ± 1.5	905.6 ± 10	909.7 ± 39.6	384.1 ± 13.9	926.9 ± 36.7
100N	890.8 ± 22.2	141.9 ± 6.1	913.3 ± 15.1	895.0 ± 63.6	359.3 ± 43.9	906.8 ± 67.8
50S	885.2 ± 21.9	139.5 ± 5.4	913.4 ± 16.6	856.7 ± 58.8	287.5 ± 24.5	813.8 ± 61.3
50N	897.3 ± 7.04	148.2 ± 7.4	908.4 ± 9.8	801.8 ± 35.9	268.9 ± 14.9	793.0 ± 40.1
10S	891.8 ± 21.9	151.3 ± 3.9	901.1 ± 15.1	938.2 ± 20.1	366.5 ± 17.3	961.2 ± 40.8
10N	831.3 ± 19.6	160.8 ± 31.7	864.9 ± 18.6	882.9 ± 17.8	229.6 ± 13.8	923.3 ± 25.3
**Suppl. form effect**						
Nanoparticles	873.2 ± 12.8	150.3 ± 10.3	895.5 ± 10.2	859.9 ± 25.9	322.6 ± 18.7	874.4 ± 30.4
Standard	888.6 ± 9.4	140.3 ± 3.4	906.7 ± 7.9	901.5 ± 24.5	346.0 ± 16.1	900.6 ± 31.2
**Dose effect**						
100%	889.8 ± 11.5	135.9 ± 3.7	909.4 ± 8.5	902.4 ± 34.8	371.7 ^a^ ± 21.8	916.8 ^a,b^ ± 35.9
50%	891.3 ± 9.7	143.8 ± 4.5	910.9 ± 9.0	829.2 ± 33.6	278.2 ^b^ ± 13.7	803.4 ^b^ ± 34.1
10%	861.6 ± 17.8	156.1 ± 14.9	883.0 ± 13.5	910.4 ± 16.2	353.1 ^a^ ± 11.4	942.3 ^a^ ± 23.4
	*p*-value					
**Source of variation**						
Suppl. Form	0.3	0.38	0.37	0.25	0.24	0.51
Dose	0.19	0.36	0.13	0.14	0.0024	0.02
Suppl. form × Dose	0.11	0.99	0.33	0.86	0.98	0.98

* 100S: 100% of the dose in a standard form; 100N: 100% of the dose in a nanoparticle form; 50S: 50% of the dose in a standard form; 50N: 50% of the dose in a nanoparticle form; 10S: 10% of the dose in a standard form; 10N: 10% of the dose in a nanoparticle form; ^a,b^ Means in the same column with different letters are significantly different (*p* < 0.05) separately between the group, between the species and between the additives.
